# The Role of OCT Angiography in the Assessment of Epiretinal Macular Membrane

**DOI:** 10.1155/2021/8866407

**Published:** 2021-03-24

**Authors:** Daniela Bacherini, Francesco Dragotto, Tomaso Caporossi, Chiara Lenzetti, Lucia Finocchio, Alfonso Savastano, Maria Cristina Savastano, Francesco Barca, Martina Dragotto, Lorenzo Vannozzi, Francesco Nasini, Francesco Faraldi, Stanislao Rizzo, Gianni Virgili, Fabrizio Giansanti

**Affiliations:** ^1^Department of Neurosciences, Psychology, Drug Research and Child Health Eye Clinic, University of Florence, AOU Careggi, Florence 50139, Italy; ^2^UOC Oculistica, Fondazione Policlinico Universitario A. Gemelli IRCCS, Rome, Italy; ^3^Centro Italiano Macula, Rome 00195, Italy; ^4^Livorno Hospital, Eye Clinic, Livorno, Italy; ^5^Torino, Eye Clinic, ASL Torino 5, 10024 Turin, Italy; ^6^Università Cattolica Del Sacro Cuore, Rome, Italy; ^7^Consiglio Nazionale Delle Ricerche, Istituto di Neuroscienze, Pisa, Italy

## Abstract

**Background:**

The aim of this observational study is to assess pre- and postoperative retinochoroidal vascular changes in patients undergoing epiretinal macular membrane (ERM) surgery by using optical coherence tomography angiography (OCTA).

**Materials and Methods:**

23 eyes affected by ERM and those which underwent phacovitrectomy associated with ERM peeling were enrolled. They were evaluated using structural OCT and OCTA before surgery and at 1, 3, and 6 months postoperatively.

**Results:**

We found a statistically significant (*p* < 0.05) increase in the superficial capillary plexus vessel density (VD) from baseline to the 6-month follow-up. We observed a large increase in both the perfusion density (PD) and the VD of the deep capillary plexus between baseline and the 6-month follow-up (*p* < 0.001). A significant decrease in the VD and PD of the choriocapillaris (CC) from baseline to the 1^st^ month and a significant increase in CC perfusion density at the 6-month follow-up compared to the preoperative value were revealed. The FAZ area and perimeter after surgery significantly increased during the follow-up (*p* < 0.001) at baseline retinal and choroidal plexi with a lower PD or VD correlated with worse visual acuity (*p* < 0.05 for all plexi). At baseline and at the 1-month follow-up, a significant correlation was found with the FAZ area and the FAZ perimeter: a smaller FAZ area or a smaller FAZ perimeter was correlated to a lower visual acuity. Before surgery, negative correlations (*p* < 0.05) were found between the Govetto ERM stages and perfusion density of the SCP and the DCP and between the Govetto stages and vessel density of the DCP.

**Conclusions:**

In our study, OCTA detected vascular alterations induced by the presence of the ERM, allowing several correlations with functional data. In these patients, OCTA may be useful to add new potential surgical prognostic factors.

## 1. Introduction

Epiretinal membrane (ERM) is a pathological condition characterized by the constitution of a fibrocellular layer over the internal limiting membrane (ILM) surface due to fibroblast proliferation after an anomalous posterior vitreous detachment [1]. ERM exerts two forces that stress and distort the retina: centripetal contraction and anteroposterior traction; the contraction of the epiretinal membrane is responsible for an additional thickening, puckering, folding, or detachment of the retinal layers, alongside with a vascular distortion [2]. Pars plana vitrectomy (PPV) associated with membrane peeling is the standard treatment for this kind of disorder, but postoperative visual prognosis is often unpredictable despite optimal and reproducible anatomical outcomes [3, 4].

Several studies have been conducted to identify markers and signs to predict postoperative visual function [5, 6]; in particular, the introduction of spectral domain optical coherence tomography (SD-OCT) has provided a powerful tool in the diagnosis and comprehension of the natural history and pathophysiology of ERMs, shifting attention from the outer retina to the inner layers [7, 8].

The retinal function also relies on vascularization, but studying ERM can be difficult and complex due to the invasiveness of fluorescein angiography; additionally, the lack of quantitative data and the visualization “en bloc” of the retinal vascular meshwork using fluorescein angiography make it an unsuitable method to evaluate ERM.

Optical coherence tomography angiography (OCTA) is a fast and noninvasive technique which allows the detailed visualization of each retinal plexus without a dye injection.

Thanks to its easy handling and safety, it can also be used to study retinal pathologies without vascular etiologies such as vitreoretinal syndromes. The in-built software permits operators to quantify the vascular alteration which can be correlated to functional parameters, such as visual acuity.

The aim of this observational cross-sectional study is to assess early retinal vascular changes in patients undergoing ERM surgery using OCTA.

## 2. Materials and Methods

This observational noncomparative cross-sectional study enrolled 21 patients (23 eyes) affected by ERM who underwent phacovitrectomy associated with ERM peeling and internal limiting membrane (ILM) peeling and who were referred to the Eye Clinic (Careggi Hospital, Florence, Italy) from September 2016 to July 2017. They were evaluated using OCT and OCTA before and after surgery.

The inclusion criteria were a diagnosis of ERM detected with B-scan OCT and adequate OCTA image quality to calculate the vessel density and the area of the foveal avascular zone (FAZ) in the superficial and deep vascular plexi (SVP and DVP, respectively) in the choroid and choriocapillaris. Exclusion criteria were opacities that interfered with the acquisition of OCT and concomitant diseases such as diabetic retinopathy, vein or artery occlusion, and glaucoma.

This study adhered to the tenets of the current version of the Declaration of Helsinki (52nd WMA General Assembly, Edinburgh, Scotland, October 2000), and written informed consent was obtained from all patients prior to participation in the study. Approval from The Institutional Review Board/Ethics Committee was obtained.

All patients underwent a baseline ophthalmic examination including medical and ocular history, family medical history, measurement of best-corrected visual acuity (BCVA) using the early treatment retinopathy diabetic study (ETRDS) chart, slit-lamp examination of the anterior and posterior segments, measurement of intraocular pressure, dilated fundus examination, and axial length measurement with noncontact partial coherence laser interferometry (IOL Master, version 3.01; Carl Zeiss Meditec, Jena, Germany), B-scan OCT, and OCTA. Patients were evaluated at 1, 3, and 6 months postoperatively. In cases with loss of visual acuity or the development of new symptoms such as scotoma or metamorphopsia, patients were recalled earlier than the standard follow-up date. The RS-3000 Advance 2 spectral domain OCT (NIDEK Co. Ltd., Gamagori, Japan) was used to acquire OCTA and end face images in all eyes. This device uses an 880 nm wavelength with a scanning speed of 53,000 A-scans/sec. A 3 mm × 3 mm (256 × 256 scan points) scanning pattern was performed in all eyes. All scans were centered on the fovea based on the live scanning laser ophthalmoscopy (SLO) image. All B-scans were performed 8 times and averaged for a higher sensitivity. A real-time SLO-based active eye tracker was used to compensate for eye movement during image acquisition. In all cases, the SLO image was captured prior to OCTA analysis. Low-quality OCTA images, severe artifacts due to poor fixation, or cases of failed automatic layer segmentation were excluded from analysis. Images were reviewed by two investigators (DB and MD) for segmentation accuracy, as Bontzos et al. found a 14-fold increased risk of motion artifact occurrence in the ERM patients, correlated with the disease severity, mostly in interior plexiform and in the ILM layers [9]. High myopic or hyperopic eyes were excluded to avoid bias in the vascular density measurement.

The default RS-3000 Advance 2 AngioScan software has been used (%) to evaluate the preoperative and postoperative vessel density (defined as the percentage of the total area occupied by vessels) and perfusion density (defined as the total area of perfused vasculature per unit area in a region of measurement).

To combine a vessel density map, images are first binarized in order to separate vessels (white) from what is not vessels (black), and then, all vessels are shrunk to 1-pixel thickness, making these mapped vessels thickness independent; finally, the sum of linear lengths of vessels per mm^2^ is then calculated point by point.

**Figure 1:**
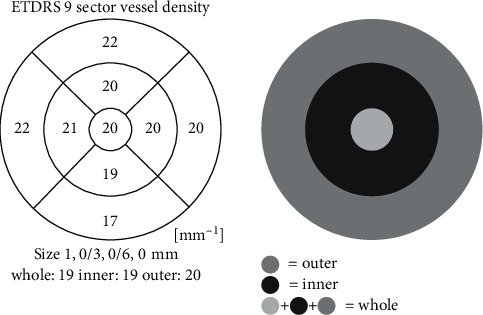
Sector vessel density.

To design a perfusion density map, again images are first binarized in order to separate vessels (white) from what is not vessels (black), but vessels are not later shrunk so as to make these mapped vessels thickness dependent; in order to map the vascularization, a percentage is calculated, indicating the vascularized tissue in the 11 × 11 pixels square centered on the concerned pixel. The spatial division in inner, outer, and whole segments is explained in Figure 1.

**Figure 2:**
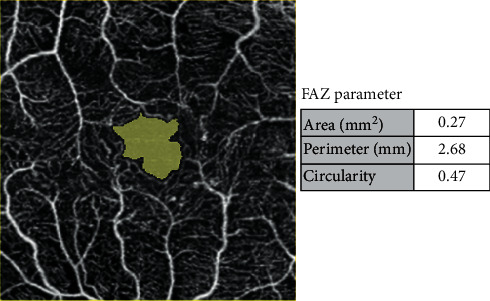
FAZ parameters.

In addition, FAZ area, perimeter, and circularity (an index that is equal to 1 when the FAZ shape is a circle) were automatically calculated by the in-built software (Figure 2).

B-scan OCT measurements included central retinal thickness (CRT) and outer nuclear layer (ONL) thickness; a preoperative qualitative analysis was conducted on each structural OCT scan to evaluate the stage of the ERM according to Govetto OCT classification, the presence of intraretinal cysts pre- and postoperatively, and the restoration of the foveal pit during follow-up examination.

All eyes underwent a standard 25-gauge 3-port pars plana vitrectomy with a wide-angle noncontact viewing system (Resight®; Carl Zeiss Meditec AG, Jena, Germany) using the Constellation Vision System (Alcon Laboratories Inc., Fort Worth, TX, USA). Brilliant Blue G (Brilliant Peel®, Fluoron GmbH, Ulm, Germany) was used to stain and peel the ILM.

A complete vitrectomy was performed, and peripheral retinal photocoagulation was carried out in eyes with retinal tears or holes. Fluid-gas exchange was then performed, followed by tamponade.

A chart review was performed to collect data on visual acuity, VD of the SVP and DVP, CRT, outer nuclear layer thickness, FAZ area perimeter, and circularity preoperatively and at 1, 3, and 6 months postoperatively. Statistical analysis included descriptive statistics of patient demographics and comparative analysis. All statistical analyses were performed using Stata. Descriptive statistics are reported as mean ± standard deviation (SD).

One-way analysis of variance (ANOVA) with repeated measurements was performed to determine the mean changes at each follow-up. The mean of each variable was compared to the baseline data.

The Spearman correlation coefficient was used to evaluate the correlation between the different parameters: a *p* > 0.05 was considered statistically significant.

## 3. Results

The study sample included 23 eyes of 21 patients (10 (48%) females and 11 (52%) males); 10 (43%) right eyes and 13 (57%) left eyes underwent surgery. The mean age was 74.38 ± 6.33 years. All the data collected are shown in Tables 1–3.

We observed a significant progressive improvement in visual acuity from baseline to the 6-month follow-up.

Concerning the superficial capillary plexus vessel density (SCPVD), we found a statistically significant (*p* < 0.05) increase from baseline to the 6-month follow-up (R: 0.698, *p* < 0.05). We did not observe statistically significant changes in the superficial capillary plexus perfusion density (SCPPD) from baseline to the postoperative follow-up.

By measuring the perfusion density and vessel density of the deep capillary plexus (DCP) before and after surgery, both were observed to have a positive trend. In fact, we observed a large increase in both the PD and the VD of this plexus between baseline and the 3-month follow-up and between baseline and the 6-month follow-up (DCPPD: 3^rd^ month R, 5.322 with *p* < 0.001 and 6^th^ R, 6.009 with *p* < 0.001; DCPVD 3^rd^ month R, 2.105 with *p* < 0.001 and 6^th^ month R, 0.614 with *p* < 0.001).

Regarding the VD and PD of the choriocapillaris plexus (CC), we found a similar positive trend. We observed a decrease in the VD and PD of the CC from baseline to the 1^st^ month (CCPD: 1^st^ month R, −2.173 with *p* < 0.05; CCVD: at the 1^st^ R, −0.884 with *p* < 0.05). We found a significant increase in CC perfusion density at the 6-month follow-up compared to the preoperative value (CCPD: R, 2.478 with *p* < 0.05). Regarding the choroidal plexus, the trend of perfusion density and vessel density was similar to that of the choriocapillaris plexus. In fact, statistically significant data (*p* < 0.05) show a decrease in R compared to baseline (CHPD: R at the 1-month −5.911 with *p* < 0.001 and CHVD: R at the 1-month follow-up was −2.270 *p* < 0.001).

Regarding the FAZ area and perimeter after surgery, we found a gradual enlargement during the follow-ups (*p* < 0.05) but the values at the 6^th^ month remained lower than at baseline (area: R at 6-month follow-up, −0.083 with *p* < 0.001; perimeter: R at 6-month follow-up, −0.417 with *p* < 0.001).

Linear correlations were found between BCVA and OCTA parameters; at baseline retinal and choroidal plexi with a lower perfusion or vessel density of the retinal and choroidal plexi correlated with worse visual acuity (SCPPD R: −0.2783 with *p* < 0.05; DCPPD R: −0.2972 with *p* < 0.05; CHPD R: −0.4217 with *p* < 0.05; DCPVD R: −0.4181 with *p* < 0.05; CHVD R: −0.4700 with*p* < 0.05). Regarding visual acuity, a correlation (*p* < 0.05) was also found with the FAZ area and the FAZ perimeter (FAZ area R: −0.2705; FAZ perimeter R: −0.3492). We observed that a smaller FAZ area or a smaller FAZ perimeter was correlated to a lower visual acuity. At the 1-month follow-up, BCVA remained significantly correlated (*p* < 0.05) to the FAZ area and the FAZ perimeter (R: −0.34 and −0.28, respectively), meaning that a larger FAZ, measured by its area or its perimeter, is associated with better visual acuity.

At the 3-month follow-up, BCVA had significant correlations (*p* < 0.05) with the choroidal plexus perfusion density (R:−0.3962) and the vessel density of the same plexus (R: −0.3095), the same that were present at baseline: a better VA correlated to a higher perfusion in the choroid or greater vessel concentration. The BCVA was also significantly (*p* < 0.05) correlated to the FAZ circularity (R:−0.5342), highlighting that a restoration of the foveal circular shape could positively affect a patient's VA. At 6 months after surgery, the BCVA maintained a significant inverse correlation only with the vessel density of the SCP (R: 0.5103), meaning that, at this time, as the vessel density of the superficial capillary plexus increased, visual acuity decreased. Significant negative correlations (*p* < 0.05) were found between the 3-month follow-up BCVA and the perfusion density of the choroidal plexus and the FAZ circularity both at baseline (CHPD R: −0.2757; FAZ circularity R: −0.3908), suggesting that patients with better visual acuity after 3 months are those who had better conservation of the foveal circularity and better perfusion in the choroidal plexus at baseline.

We classified each ERM using the Govetto [7] OCT classification, and then, we correlated the different ERM stages with the OCT and OCTA parameters. We found some significant correlations both at baseline and at the 3-month follow-up.

At baseline, we found a significant negative correlation (*p* < 0.05) between the Govetto stages and BCVA (R: 0.5342), meaning that as the Govetto stages progress, BCVA decreases. Before surgery, negative correlations (*p* < 0.05) were found between the Govetto stages and perfusion density of the SCP and the DCP and between the Govetto stages and vessel density of the DCP (SCPPD R:−0.2544; DCPPD R:−0.3788; DCPVD R:−0.4337). Therefore, a higher ranking in this classification is associated with a reduction in perfusion density of the SCP and the DCP and a reduction in vessel density of the DCP. At baseline, there was a significant positive correlation (*p* < 0.05) between the Govetto stages and vessel density of the choriocapillary plexus (CCVD R:0.2857). Significant negative correlations (*p* < 0.05) were found between the stages and the choroidal perfusion and vessel density, and the perfusion density of the outer retinal choriocapillaris (CHPD R: −0.4809; CHVD R: −0.5030; ORCCPD R:−0.4003). Regarding the FAZ area and the FAZ perimeter, we found significant negative correlations (*p* < 0.05) between these parameters and the Govetto stages (FAZ area R:−0.5741; FAZ perimeter R: −0.4908), meaning that a smaller FAZ area or FAZ perimeter is associated with a higher degree in the ERM classification. Finally, we observed a significant positive correlation (*p* < 0.05) between the Govetto stages and the CRT (crt R: 0,6709), which means that a greater thickness of the central retina corresponds to a higher ranking of Govetto's stages.

Concerning the 3-month follow-up, significant negative correlations (*p* < 0.05) were found between the Govetto stages and perfusion and vessel density of the DCP (DCPPD R: −0.3083; DCPVD R: −0.3579). We also observed significant negative correlations (*p* < 0.05) between the Govetto stages and the perfusion density of the choroidal plexus (CHPD R: −0.2953) and between the Govetto stages and FAZ circularity (FAZ circularity 3 R: −0.3098). At the 3-month follow-up, we also observed positive correlations (*p* < 0.05) between the Govetto stages and choriocapillaris plexus vessel density (CCVD R: 0.3856), the outer retinal choriocapillaris perfusion density (ORCCPD R: 0.3231), and outer retinal choriocapillaris vessel density (ORCCVD R: 0.2855). A compromising ERM at baseline can affect retinal vasculature over time even after its removal.

We made a comparison between the ONL at baseline and the restoration of the foveal pit at the 3-month follow-up. At the 1-month follow-up, the restoration of the foveal pit was observed in 48% of the patients. We found out that the patients with higher preoperative ONL thickness had lower probability of restoration of the foveal pit (*p* = 0.0015).

We compared the patients' ONL at baseline with the patients' data at the 3-month follow-up, and we found various statistically significant correlations (*p* < 0.05). Specifically, ONL at baseline is negatively correlated with CHVD at 3 months (R: −0.3157). ONL at baseline is also positively correlated with the 3-month follow-up FAZ circularity (R: 0.3978) and CRT (R: 0.7475).

**Figure 3:**
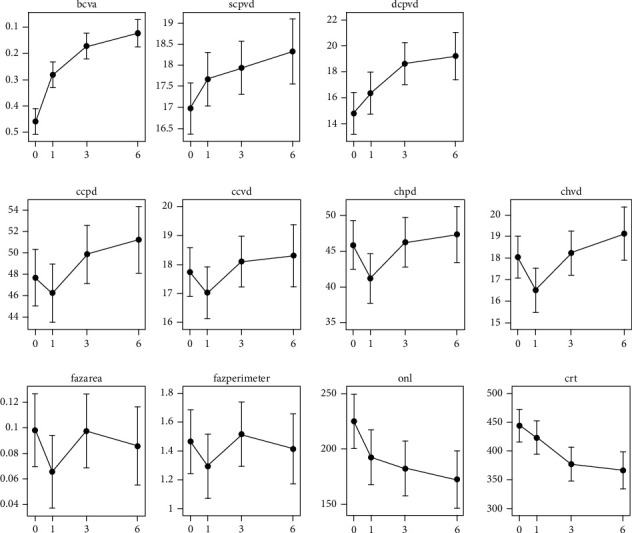
Progression graphs. On the abscissa, the time expressed in months is presented.

In Figure 3, progression graphs are shown.

## 4. Discussion

The presence of an ERM, depending on its thickness and traction force, causes a retinal distortion, in particular in the macular region. By means of fundus oculi examination in patients affected by ERM, an increase in vascular tortuosity and the retinal contraction itself can easily be seen but are difficult to quantify. The use of OCTA has introduced the measurement of different parameters which can be used in this kind of disorder to assess what is distorted and how much the ERM contractions affect the retina.

In this study, a significant increase in retinal and choroidal vessel and perfusion density was found after surgery. Literature highlights that the SCP is greatly affected in ERM-affected eyes [10]. Mastropasqua et al. [11] found that the preoperative perfusion density and vessel density were statistically lower than that in the control group; it can be hypothesized that partial capillary subocclusion occurred related to ERM presence, thus causing flow impairment in the foveal region. However, one of the other possibilities is that actual vessel and perfusion density were not reflected in preoperative OCTA image because the capillaries were folded with ERM, and further studies are needed to confirm those hypotheses.

We found a significant progression and increase in SCP vessel density from baseline to the 6-month follow-up, probably because it is more sensitive to a reopening of little vessels that were suboccluded in the preoperative period.

**Figure 4:**
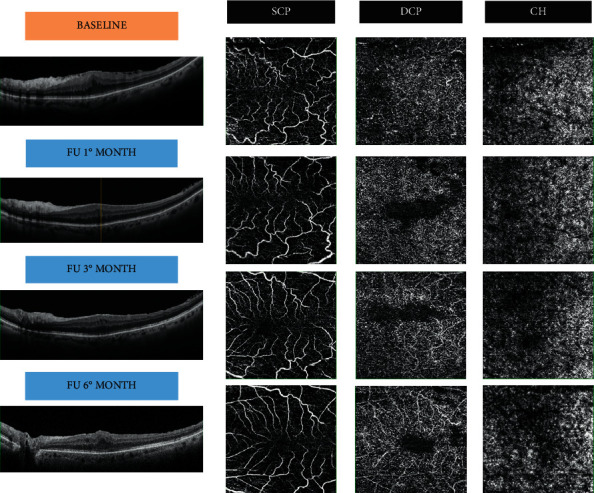
Vascular changes after ERM removal in VD. FU: follow-up; SCP: superficial capillary plexus; DCP: deep capillary plexus; CH: choroid.

Regarding the DCP, a significant increase can be seen during the follow-ups both in vessel and in perfusion density, as little capillaries in this plexus can easily be distorted and occluded by ERM tractional forces and its removal causes a gradual reopening and blood flow increase that lasts several months. Lin et al. [12] observed some focal perfusion areas as irregularly shaped patches in DCP, correlated to hypofluorescent areas in fluorescein angiography. As explanation of this phenomenon, they proposed that the tractional stretching forces exerted on the retina empty these capillary patches without affecting the vascular wall; after surgery, a reperfusion with a reappearance of the vascular net can be found (Figure 4).

Rommel et al. proposed that choroidal and choriocapillary networks are affected by ERMs that the impairment in the superficial and the deep retinal plexi may subsequently influence the microvasculature in the choriocapillaris [13].

In our investigation, both these plexi have a peculiar progression: from baseline to the first month, we can see a reduction in vessel and perfusion density and, postoperatively, a significant increase. Li et al. [14] have explained the variations found as retinal blood flow improvement after vitrectomy, followed by retinal arteriolar saturation which increases due to the removal of vitreous body, which could reduce the retinal oxygen consumption and allow oxygen diffusion and transport from the anterior segment. There was no parallel change in venous saturation which suggests that it was possibly caused by better oxygen use during tissue repairment during this period.

Regarding the FAZ area, its behavior is peculiar: a significant reduction was found after peeling at the 1-month follow-up, but later, a gradual enlargement was observed. Some papers [15, 16] suggest that a reduced FAZ area increase after surgery can be caused by a collateral effect of the ILM peeling. One explanation is that the ILM may have some intrinsic forces stretching the retina centrifugally, and its peeling may remove such forces leading to a centripetal movement. The second hypothesis is that the structural changes in the Muller cells, caused by damage during ILM removal, may influence the inner retinal movement. These cells also act as a scaffold that stretches the macula outwards, the removal of their footplates which are anchored to the ILM stop this action, and the macula may move inwards. Those hypotheses have, although, to be confirmed by further studies with a larger number of participants.

At baseline, we found that retinal and choroidal plexi with lower perfusion or vessel density are correlated to worse visual acuity, highlighting that ERM causes a full thickness retinal and choroidal impairment. Gradually, these correlations disappear and only the SCP vessel density remains significantly related to visual acuity after 6 months. Probably, as the innermost retinal layers are the most affected [7] and ERM surgery can also damage this region [17], the SCP vascular network could be an indirect sign of any injury received. Also, the FAZ area and the FAZ perimeter are correlated to BCVA at baseline. Some authors [18, 19] suggest that a vessel crowding in the FAZ area impairs visual acuity because of light interference in a clear optical zone when perifoveal vessels are pulled inward and prevent light reaching photoreceptors, dispersing light before it reaches outer retinal layers. Interestingly, BCVA at the 3-month follow-up and the FAZ circularity calculated at baseline are correlated, suggesting that patients with better visual acuity after 3 months are those who had better conservation of foveal circularity; the FAZ circularity may quantify the disruption of the terminal capillary ring at the fovea, and it may be a better measurement to assess the degree of microvascular damage at the FAZ, which is more related to vision [20].

The 3-month BCVA had direct correlation to preoperative choroidal perfusion density; tractional forces can also affect the choroidal layers [13], especially the subfoveal choroidal thickness (SFCT) and the perfusion of Haller's layer (HLP) decreasing from morning to afternoon, before slightly increasing again in the evening. These variations differ from healthy control patients where the thinning is during the daytime and the thickening during the night, and the explanation proposed is that flow alteration in the superficial and deep capillary plexi may subsequently influence microvasculature in the choriocapillaris.

Other studies have proposed a choroidal involvement in ERM natural history and the correlation of this vascular network with visual acuity [21]: the foveal region obtains most of its oxygen from the choroid; in this macular stress condition, we can hypothesize that higher values of perfusion can provide better retinal oxygenation and subsequently consent a better functional outcome during follow-up.

In our study, we have analyzed the possible correlations that the new ERM, OCT-based, Govetto classification could have with the various parameters studied using OCTA both before and after surgery.

**Figure 5:**
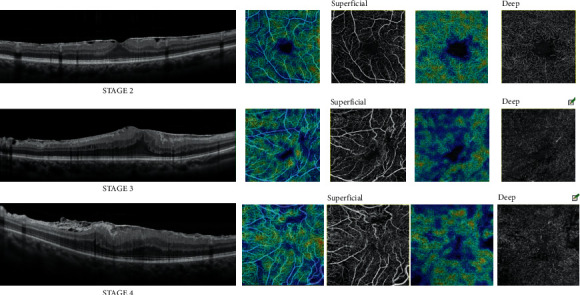
Vessel density and perfusion density correlated to ERM status.

Before surgery, the Govetto stages had negative correlations with the perfusion density and the vessel density of the DCP and with the perfusion density of the SCP. Consequently, we may hypothesize that, in the preoperative period, a higher ranking in the Govetto classification is associated not only with lower visual acuity but also with a significant alteration in retinal microcirculation. These correlations between the Govetto stages and the vessel density and perfusion density of the DCP have also been found at the 3-month follow-up, meaning that a preoperative Govetto stage 3 or 4 epiretinal macular membrane is associated in the postoperative period with a worse restoration of retinal circulation (Figure 5).

In our study, we also observed significant negative correlations at baseline between the Govetto stages and the FAZ area and FAZ perimeter, meaning that a smaller FAZ area or FAZ perimeter are associated with a higher rank in the ERM Govetto classification.

We found significant correlations between preoperative ONL thickness and the restoration of the foveal pit after 3 months. The ONL has recently been seen as one of the most distorted retinal layers in the natural history of ERMs [7]. We found that a thicker ONL is associated with a more infrequent restoration of the foveal pit. A hypothesis that we can put forward is that an abnormal elongation of this layer could be an indirect sign of a loss of flexibility of the full thickness retina; further studies are needed to assess whether there is a thickness measurement over which it is hard to achieve a restoration of the normal anatomical retinal shape after surgery.

Our study has several limits: the sample size is relatively small, and a larger number of patients should be evaluated to confirm our results. The follow-up period was relatively short, and further prospective randomized studies with longer observation periods are necessary to confirm these results.

We have revealed the presence of intraretinal microcysts in the extrafoveal region in 2 patients both at the 1^st^ month of follow-up and at the 3^rd^ although they have not interfered with proper layer segmentation and the FAZ calculation and measurement. For this reason, we do not believe that the data obtained could be related to the presence of a pseudophakic edema. Our hypotheses have the necessity to be confirmed by further and larger studies.

## 5. Conclusions

The purpose of our study was to understand how preoperative alterations can affect the postoperative visual results. The most significant quantitative alterations of retinal vascularity involve the FAZ and the retinal and choroidal plexi and are correlated to ERM severity, measured using the Govetto classification.

To date, the exact timing of ERM surgery is not strictly defined. Generally, a worsening of a patient's symptoms, such as an increase in metamorphopsia or a decrease in visual acuity, leads to surgery. These factors, however, are not standard and quantifiable and sometimes occur late in the disease, so the choice of surgical timing is still very arbitrary.

OCTA could be useful to evaluate retinal circulation in a rapid and noninvasive way and can be performed at the same time as the structural OCT necessary for the diagnosis and classification of ERM. Since vitreoretinal surgery is elective, further studies are necessary to define the role and clinical usefulness of OCTA in the routine assessment of patients affected by ERM and to help establish the correct surgical timing (Table 1)

## Figures and Tables

**Table 1:** Patients' demografical and anatomical data.

Follow-up	Age (year)	Axial length (mm)	Best corrected visual acuity (logMAR)	Govetto classification (eyes)				Central retinal thickness (*μ*m)	Outer nuclear layer thickness (*μ*m)
Baseline	75,27 ± 6,33	23.61 ± 0.78	0,47 ± 0,19	1:4	2:9	3:7	4:3	445,91 ± 78,86	230 ± 65,26
1^st^month			0,28 ± 0,15^∗∗^					432,67 ± 90,56 ^*∗*^	201,45 ± 59,05
3^rd^month			0,16 ± 0,12^∗∗^					384,35 ± 59,23	192,37 ± 58,04
6^th^month			0,11 ± 0,12^∗∗^					375,18 ± 62,03	179,45 ± 53,25 ^*∗*^

^*∗*^ *p* < 0.05; ^*∗∗*^*p* < 0.01

## Data Availability

The data used to support the findings of this study are available from the corresponding author on reasonable request.

## Supplementary Materials

Supplementary Materials Table 1: vessel density parameters. Table 2: perfusion density parameters. Table 3: FAZ parameters.
