# Supplementary material for: The Role of OCT Angiography in the Assessment of Epiretinal Macular Membrane
Source: J Ophthalmol. 2021 Mar 24;2021:8866407. doi: 10.1155/2021/8866407 (PMC8012119; doi:10.1155/2021/8866407)
Supplement: Supplementary Materials — Table 1: vessel density parameters. Table 2: perfusion density parameters. Table 3: FAZ parameters. [file 8866407.f1.docx]

*Table 2*

|  | Superficial capillary plexus (%) | | | Deep capillary plexus (%) | | | Choroidal plexus (%) | | | Choriocapillary plexus (%) | | | |
| --- | --- | --- | --- | --- | --- | --- | --- | --- | --- | --- | --- | --- | --- |
| Vessel density | whole | inner | outer | whole | inner | outer | whole | inner | outer | whole | inner | outer |  |
| Baseline | 16,18 ± 2,32 | 16,96 ± 2,53 | 17,26 ± 2,26 | 14,52 ± 4,8 | 15,48 ± 5 | 15 ± 5,36 | 17,52 ± 1,95 | 17,48 ± 2,02 | 19,35 ± 2,06 | 17,65 ± 2,06 | 17,35 ± 2,01 | 18,3 ± 3,04 |  |
| 1^st^ month | 17,65 ± 1,84 | 17,8 ± 1,61 | 17,55 ± 2,89 | 16 ± 4,97 | 16 ± 5,03 | 17,05 ± 6,06 | 15,89 ± 4,71* | 16,4 ± 4,56 | 17,4 ± 5,14 | 17,25 ± 3,4* | 16,35 ± 2,72 | 18 ± 3,88 |  |
| 3^rd^ month | 17,8 ± 1,82 | 17,95 ± 1,54 | 17,95 ± 2,37 | 17,6 ± 3,25** | 18,6 ± 3,45 | 19,15 ± 3,67 | 17,5 ± 2,16 | 17,85 ± 2,34 | 19,2 ± 2,88 | 18 ± 2,29 | 17,2 ± 2,09 | 19,1 ± 2,99 |  |
| 6^th^ month | 17,81 ± 1,68* | 17,81 ± 1,08 | 18,54 ± 1,69 | 18,45 ± 2,25** | 19,18 ± 2,79 | 20,45 ± 3,47 | 16,64 ± 1,50 | 18,54 ± 1,81 | 20,63 ± 2,42 | 18,18 ± 2,13 | 17,63 ± 1,69 | 19,18 ± 3,25 |  |

*Table 3*

|  | Superficial capillary plexus (%) | | | Deep capillary plexus (%) | | | Choroidal plexus (%) | | | Choriocapillary plexus (%) | | |
| --- | --- | --- | --- | --- | --- | --- | --- | --- | --- | --- | --- | --- |
| Perfusion density | whole | inner | outer | whole | inner | outer | whole | inner | outer | whole | inner | outer |
| Baseline | 49,7 ± 5,7 | 50,74 ± 6,4 | 50,7 ± 5,99 | 36,39 ± 13,84 | 40,17 ± 14,94 | 35,97 ± 14,5 | 44,57 ± 6.9 | 45,74 ± 7,78 | 48,26 ± 7,29 | 47,6 ± 5,96 | 48,13 ± 6,6 | 47,6 ± 7,61 |
| 1^st^ month | 50,3 ± 5,64 | 52 ± 5,08 | 49,6 ± 7,76 | 40,15 ± 13,78 | 43,4 ± 14,27 | 41,4 ± 15,63 | 40,05 ± 13,04* | 41,8 ± 12,90 | 43,4 ± 14,55 | 46,55 ± 9,64* | 46,35 ± 9,13 | 47,6 ± 11,06 |
| 3^rd^ month | 50,85 ± 3,8 | 52,85 ± 2,92 | 50,25 ± 5,33 | 43,75 ± 8,84** | 47,75 ± 9,69 | 45,15 ± 9,39 | 43,7 ± 6,89 | 45,2 ± 7,65 | 48,6 ± 9,95 | 49,4 ± 6,05 | 48,85 ± 6,41 | 50,85 ± 7,42 |
| 6^th^ month | 52,18 ± 10,08 | 53,54 ± 1,21 | 52,18 ± 2,64 | 46,9 ± 5,56** | 49 ± 5,53 | 48,18 ± 5,56 | 46,54 ± 3,17 | 47,36 ± 3,81 | 51,90 ± 4,76 | 51,45 ± 3,83* | 51,90 ± 2,02 | 52,36 ± 6,8 |

*Table 4*

| FAZ | Area (mm^2^) | perimeter (mm) | Circularity |
| --- | --- | --- | --- |
| Baseline | 0,1 ± 0,9 | 1,48 ± 0,66 | 0,48 ± 0,13 |
| 1^st^ month | 0,05 ± 0,04** | 1,2 ± 0,46** | 0,52 ± 0,28 |
| 3^rd^ month | 0,08 ± 0,06** | 1,43 ± 0,52** | 0,47 ± 0,11 |
| 6^th^ month | 0,07 ± 0,06** | 1,31 ± 0,63** | 0,5 ± 0,07 |
